# In vitro screening of UGT2B10 in silico prioritized putative ligands from drugs used in the pediatric hematopoietic stem cell transplantation setting

**DOI:** 10.1002/prp2.70011

**Published:** 2024-11-29

**Authors:** Yahia Bennani, Khalil Ben Hassine, Muhammed Gencaslan, Mary Boudal‐Khoshbeen, Caroline Samer, Marc Ansari, Youssef Daali, Chakradhara Rao Satyanarayana Uppugunduri

**Affiliations:** ^1^ Division of Clinical Pharmacology and Toxicology Geneva University Hospitals Geneva Switzerland; ^2^ Geneva Lausanne School of Pharmacy University of Geneva Geneva Switzerland; ^3^ Cansearch Research Platform for Pediatric Oncology and Hematology, Faculty of Medicine, Department of Pediatrics, Gynecology and Obstetrics University of Geneva Geneva Switzerland; ^4^ Division of Pediatric Oncology and Hematology, Department of Women, Child and Adolescent University Geneva Hospitals Geneva Switzerland; ^5^ Swiss Center for Applied Human Toxicology Geneva Switzerland; ^6^ Faculty of Medicine University of Geneva Geneva Switzerland; ^7^ Department of Medical Oncology Jawaharlal Institute of Postgraduate Medical Education and Research Puducherry India

**Keywords:** acetaminophen, glucuronidation, hematopoietic stem cell transplantation, lorazepam, mycophenolic acid, sinusoidal obstruction syndrome, UGT1A4, UGT1A6, UGT2B10, voriconazole

## Abstract

UGT2B10 is a phase II drug metabolizing enzyme with limited information on its role in the metabolism of drugs, especially in the pediatric hematopoietic stem cell transplantation setting. Previously, we investigated UGT2B10's role through in silico analyses and prioritized acetaminophen (APAP), lorazepam (LOR), mycophenolic acid (MPA), and voriconazole N‐oxide (VCZ N‐oxide) for in vitro investigations. In this report, we present in vitro screening of these candidates and of voriconazole (VCZ) to assess their potential to be substrates and/or inhibitors of UGT2B10. Enzyme kinetics experiments included recombinant UGT2B10 and analytical methods based on ultra high‐performance liquid chromatography coupled to mass spectrometry (UHPLC–MS). To determine potential substrates, candidates were incubated at various therapeutically observed concentrations with recombinant UGT2B10 to identify the corresponding glucuronide metabolite. Inhibition capacity was tested using the selective probe cotinine for its glucuronidation to cotinine N‐ß‐d‐glucuronide. IC_50_ was determined for compounds exhibiting inhibition. Among the tested compounds, LOR (IC_50_ = 0.01 μM, *R* ^2^ = 0.9257) and MPA (IC_50_ = 0.38 mM, *R* ^2^ = 0.9212) exhibited inhibition potential for UGT2B10. None of the other tested compounds featured inhibition potential and none of the compounds tested exhibited metabolism through UGT2B10. Further exploration on the clinical relevance of this inhibition using modeling strategies, overlapping nature with other UGT isoforms, and screening other molecules for their inhibition potential on UGT2B10 is warranted.

**Abbreviations**
AMT: amitriptyline
APAP: acetaminophen
*C*_max_
: maximum plasma concentration in adult population
HRMS: high‐resolution mass spectrometry
LC: liquid chromatography
LOR: lorazepam
MPA: mycophenolic acid
Mo‐3‐G: morphine‐3‐ß‐glucuronide
MS: mass spectrometry
MS/MS: tandem mass spectrometry
RSD: relative standard deviation
SD: standard deviation
SOS: sinusoidal obstruction syndrome
UDPGA: UDP‐D‐glucuronide trisodium salt
UHPLC: ultra high‐performance liquid chromatography
VCZ: voriconazole

## 1 INTRODUCTION

Uridine diphosphate (UDP) glucuronosyltransferases (UGTs) superfamily is a ubiquitous group of enzymes highly expressed in the liver, to a certain extent in the gastrointestinal tract, kidneys, and contribute to the phase II metabolism of drugs. A large interindividual variability exists in the expression of this superfamily of enzymes.^1^, ^2^, ^3^ Glucuronidation accounts for 35% of the phase II metabolism with wider involvement of UGT2B family members, including UGT2B10.^1^, ^3^, ^4^, ^5^, ^6^ UGT2B10 catalyzes the N‐glucuronidation of drugs containing tertiary amines such as tricyclic antidepressants, nicotine, and its metabolite, cotinine.^7^, ^8^, ^9^

UGT2B10 is less explored, probably due to its low relative hepatic abundance (2%–7%), compared to other UGTs, for example, UGT2B7 (18%–27%) and UGT1A1 (12%–27%).^2^, ^10^, ^11^ Target peptide identification methods reported lower UGT2B10 levels in HLMs compared to UGT1A4, UGT2B7, and UGT2B15. However, UGT2B10 levels were higher than UGT2B17 and UGT1A9 isoforms.^3^ Many SNPs and alternate spliced transcripts within *UGT2B10* were reported.^10^, ^12^ UGT2B10 matures early in children and may have clinical relevance in pediatric populations. Despite its potential relevance, limited information on UGT2B10 phenotypes, substrates, inhibitors, and inducers is available.^4^ Several drugs were reported to modulate the enzymatic activity of this enzyme. For instance, fluconazole inhibits UGT2B10 and other UGT2B family members.^13^ Since UGT2B10 is matured early in life, unlike other UGTs (e.g., UGT1A6 and UGT2B17), it may play a significant role in N‐glucuronidation in pediatric patients.^4^

In an exome‐wide association study in pediatric hematopoietic stem cell transplantation (HSCT) setting for sinusoidal obstruction syndrome (SOS), a variant in *UGT2B10* (rs17146905) correlated with a higher risk of developing this pathology.^14^ SOS is a serious complication occurring in 22%–30% of pediatric patients after HSCT^15^, ^16^, while the role of UGT2B10 in relation to SOS occurrence is poorly understood. Moreover, UGT2B10 is relatively not well explored for its substrates and perpetrators. Based on the findings of the exome‐wide association study by Ansari et al.,^14^ we conducted a computational analysis to assess UGT2B10's potential involvement in metabolizing drug candidates used in hematopoietic stem cell transplantation (HSCT). We also investigated whether these candidates could potentially act as ligands for UGT2B10, as described in Robin et al.^11^

In the present in vitro study, we assessed the prioritized ligands from in silico analyses for their potential to be substrates and/or inhibitors of UGT2B10. This approach may aid the establishment of an in silico‐in vitro screening pipeline for substrates and inhibitors of UGT2B10 by combining in vitro enzymatic assays and detecting metabolites through ultra high‐performance liquid chromatography coupled to mass spectrometry (UHPLC–MS) following an in silico prioritization.

## 2 MATERIALS AND METHODS

### 2.1 Chemicals, reagents, and enzymatic systems

Acetaminophen (APAP), APAP‐glucuronide, mycophenolic acid (MPA), lorazepam (LOR), LOR‐glucuronide, amitriptyline (AMT), cotinine, morphine‐3‐ß‐glucuronide (Mo‐3‐G) and midazolam were purchased from Sigma Aldrich (Darmstadt, Germany). Cotinine N‐ß‐d‐glucuronide was purchased from Toronto Research Chemical (Toronto, Canada). MPA‐ß‐d‐glucuronide was purchased from AlsaChim (Illkirch‐Graffenstaden, France). Voriconazole (VCZ) N‐oxide was purchased from Glentham Life Sciences (Corsham, UK) and VCZ was purchased from Sigma‐Aldrich (Missouri, USA). VCZ N‐oxide‐glucuronide is not available on the market.

Recombinant UGT2B10, UGT1A6, and UGT1A4 proteins were purchased from Corning (New York, USA). The tris–HCl Buffer was purchased from Billerica MA. The UDP‐D‐glucuronide trisodium salt (UDPGA) was purchased from Carbosynth and MgCl_2_ was purchased from Sigma Aldrich (Darmstadt, Germany).

The stock concentration and solvent used to dissolve each compound are listed in Table S1. The stock solutions were aliquoted according to the experimental needs. Recombinant protein aliquots were kept at −80°C and all other reagents were stored at −20°C.

### 2.2 Analytical methods

#### 2.2.1 Quantification of cotinine N‐ß‐d‐glucuronide

An analytical method for the quantification of cotinine N‐ß‐d‐glucuronide formation from cotinine, catalyzed by UGT2B10, was developed and validated.^13^ Briefly, all samples were analyzed by UHPLC–MS/MS with an Agilent series 1100 (Waldbronn, Germany) coupled with electrospray ionization (ESI) and an API 4000 MS from AB Sciex (Canada). Positive mode with multiple reaction monitoring (MRM) scan type was used to quantify cotinine N‐ß‐d‐glucuronide from 10 to 1000 ng/mL (7 levels), in the presence of 450 ng/mL of Mo‐3‐G as internal standard. Mass transitions and mass spectrometry parameters are listed in Table S2.

Chromatographic conditions for cotinine N‐ß‐d‐glucuronide quantification were the following: a gradient elution method with a flow rate of 500 μL/min, comprising 5% ammonium formate 20 mM pH 6.5 (phase A) and 95% acetonitrile (phase B) initially, was used. Phase B was increased from 5% to 45% beginning from 2 to 5 min, then maintained at 45% for 1 min. From 6 to 6.1 min, conditions were reset to initial parameters which were maintained until the end of the analysis at 12 min. The stationary phase used was a Kinetex hydrophilic interaction liquid chromatography (HILIC) column (100 A, 2.6 μm, 2.1 × 50 mm) purchased from Phenomenex (Torrance, USA).

The analytical method was fully validated as per the current European Medicines Agency (EMA) guidelines^17^ (https://www.ema.europa.eu/en/bioanalytical‐method‐validation‐scientific‐guideline) and the FDA bioanalytical method validation guidance for industry^18^ (https://www.fda.gov/regulatory‐information/search‐fda‐guidance‐documents/bioanalytical‐method‐validation‐guidance‐industry) by evaluating its selectivity, carry‐over, linearity, accuracy, precision and stability as detailed in Text S1.

#### 2.2.2 Detection of glucuronide metabolites of UGT2B10 putative ligands

For the detection of putative glucuronide metabolites, standard and reaction samples were analyzed using the same instrument and in positive mode as for cotinine N‐ß‐d‐glucuronide quantification. The following chromatographic conditions were implemented: A gradient elution method with a flow rate of 300 μL/min, comprising 90% ammonium formate 20 mM pH 6.5 (phase A) and 10% methanol (phase B) initially, was used. From 1 to 4 min, phase A was decreased from 90% to 10%. It remained at 10% for 1 min before returning to 90% from 5 to 5.10 min and staying at 90% from 5.10 to 11 min, which marked the end of the analysis. The applied stationary phase was a Discovery HS C18 (5 μm, 15 cm × 2.1 mm; Sigma Aldrich, Darmstadt, Germany). Mass transitions are listed in Table S2.

In addition, high‐resolution mass spectrometry (HRMS) Orbitrap Exploris 120 (Thermo Fisher Scientific, Bremen, Germany) coupled with a Vanquish UHPLC system (Dionex Softron GmbH, Germering, Germany) was used to explore VCZ‐glucuronide production from VCZ via UGT2B10. Chromatographic conditions used in UHPLC‐HRMS were identical to those used in UHPLC–MS/MS. However, HRMS detection implied no fragmentation. Instead, voriconazole‐glucuronide was directly detected through its accurate m/z. The same procedure was implemented for the assessment of UGT1A4‐mediated glucuronidation of both VCZ and midazolam.^5^

### 2.3 Optimization of enzymatic assay parameters

Incubations for enzymatic assays were prepared in 100 mM tris–HCl buffer (pH 7.4) containing 5 mM cotinine (substrate), 5 mM MgCl_2_, and 0.5 mg/mL UGT2B10 enzyme. After 10 min of preincubation at 37°C, the reaction was initiated with 5 mM of UDPGA. After an optimal incubation time (experimentally determined), the reaction mixture was quenched with ice‐cold acetonitrile comprising 450 ng/mL of Mo‐3‐G. The solution was centrifuged at 10000 rpm for 10 min at 4°C and the collected supernatant was analyzed using analytical methods described. DMSO concentration (in the range of 0, 1, 2, 5, and 10% in reactions), and time of incubation (0, 10, 20, 30, 60, 90, and 120 min) were optimized using three and two times replicated experiments, respectively. Then, the Michaelis constant (*K*_m_) of cotinine with UGT2B10 was determined, and the screening of UGT2B10 inhibitors or substrates was performed. According to the literature, the *K*_m_ of cotinine ranges from 1 to 3.5 mM.^13^ However, the experimental *K*_m_ must be determined in order to choose the optimal concentration for the incubation and to avoid saturating the active sites of the recombinant UGT2B10 protein used. *K*_m_ was determined by incubating 1, 2, 5, and 10 mM of cotinine with UGT2B10 under optimal DMSO concentration (<0.5%) for the optimal time defined in earlier experiments, in three replicates.

### 2.4 Screening of UGT2B10 putative inhibitors

Initially, test compounds were screened for UGT2B10 inhibition at 5 × *C*_max_ (five times the peak clinically observed plasma concentrations in the adult population). Compounds showing a significant inhibition at 5 × *C*_max_ were further evaluated at *C*_max_ concentrations (Table 1). Compounds featuring an inhibition at *C*_max_ concentrations were subject to the determination of their half‐maximal inhibitory concentration (IC_50_). AMT, a potent UGT2B10 inhibitor,^13^, ^19^ was used at 10 μM as a positive control. Negative controls with no known/putative inhibitors were also implemented. Incubations at 5 × *C*_max_ and *C*_max_ as well as IC_50_ incubations consisted of three and five replicates, respectively. After prior screening, IC_50_ was determined for LOR and MPA using concentration ranges mentioned in Table 1.

**TABLE 1:** *C*_max_ and IC_50_ screening ranges for the four prioritized molecules.

Drug	APAP	VCZ N‐oxide	MPA	LOR
*C*_max_ value	0.05 mM^20^	0.015 mM^21^	0.08 mM^22^	0.03 μM^23^
IC_50_ screening range	—	—	*C*_max_/3–10 × *C*_max_ (8 levels)	*C*_max_/3–5 × *C*_max_ (6 levels)

### 2.5 Screening of UGT2B10 putative substrates

Putative substrates were explored using UGT2B10 enzymatic assay upon incubations at *C*_max_ concentrations, following compound concentrations reaching 10 × *C*_max_. The positive and negative controls consisted of the incubation of cotinine with UGT2B10 and the incubation of samples without the enzyme, respectively. Moreover, an additional similar incubation of each prioritized molecule was performed with UGT1A6 instead of UGT2B10 to explore the potential O‐glucuronidation of each compound (vs. N‐glucuronidation), thus comparing the implication of both glucuronidation pathways. VCZ was also incubated with UGT1A4 for 120 min to explore the formation of VCZ N‐glucuronide, in addition to a positive control for UGT1A4 with midazolam.^5^ Appropriate negative controls with no enzymes in the incubations but with cofactors were included. All the incubations were prepared in triplicates. Samples were analyzed using UHPLC–MS/MS method except for VCZ samples which were analyzed using UHPLC‐HRMS method.

### 2.6 Data analysis

The enzyme kinetics data (substrate‐to‐metabolite formation) were analyzed on GraphPad Prism 9.5.1 software to perform the Michaelis–Menten regression for *K*_m_ and IC_50_ determination. Nonlinear regression curve fitting was used to calculate *K*_m_ and IC_50_ values. Four different concentrations of cotinine using three replicates were prepared to determine its *K*_m_. Varying concentrations of the inhibitor as well as incubations without inhibitor were used for the IC_50_ determination.

## 3 RESULTS

### 3.1 Quantification of cotinine and putative ligands glucuronidation mediated by UGT2B10

Cotinine N‐ß‐d‐glucuronide formation mediated by UGT2B10 was successfully detected as illustrated in Figure S1 and the quantification method was validated with an accuracy of 91.5%–111.8% and a precision of 8.5%–11.0%. Validation results are detailed in Text S2. Glucuronide metabolites of APAP, LOR, and MPA (standards) were successfully detected. Chromatographic peaks are also illustrated in Figure S1.

### 3.2 UGT2B10 enzymatic assay parameters optimization

**FIGURE 1:**
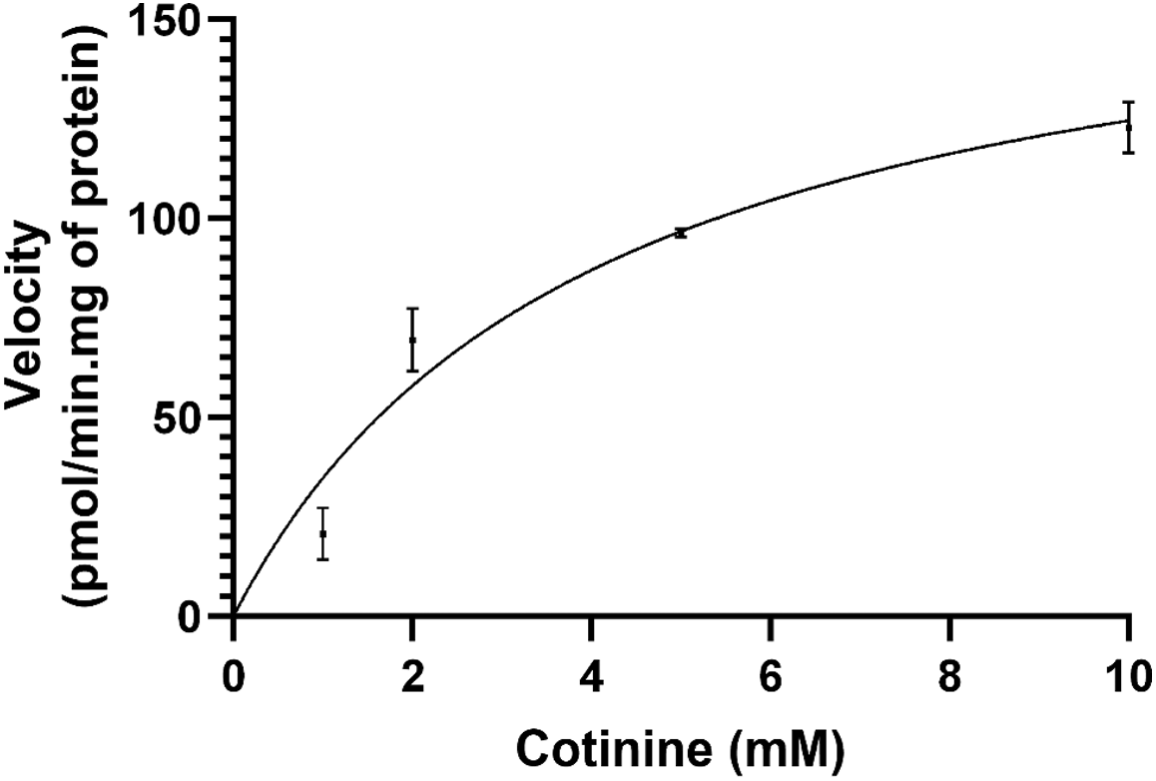
Michaelis–Menten plot of UGT2B10 enzyme activity. Increased concentrations of the substrate cotinine were used (*x*‐axis) with a fixed concentration of UDPGA (5 mM) to determine recombinant UGT2B10 (0.5 mg/mL) activity (*y*‐axis). *K*_m_ and *V*_max_ values for cotinine were determined as 4.043 mM and 175 pmol/min.mg of enzyme (*p*‐value of the fit = 0.0018) according to the curve of the graph. Each data point on the plot represents the mean of three replicates with SD.

The optimal incubation time was 90 min resulting in the optimal amount of cotinine N‐ß‐d‐glucuronide produced for analytical measurements in inhibition experiments, which exceeded 150 ng/mL (Figure S2A). DMSO concentrations used in the experiments did not exceed 0.5%, as defined by evaluating its impact on cotinine N‐ß‐d‐glucuronide formation (Figure S2B). The experimental *K*_m_ and *V*_max_ of cotinine for UGT2B10 were determined to be 4.043 mM and 175 pmol/min/mg of the enzyme (Figure 1 **)**. Cotinine was used at 4 mM concentration for the screening of UGT2B10 inhibitors.

### 3.3 Screening of UGT2B10 inhibitors

**FIGURE 2:**
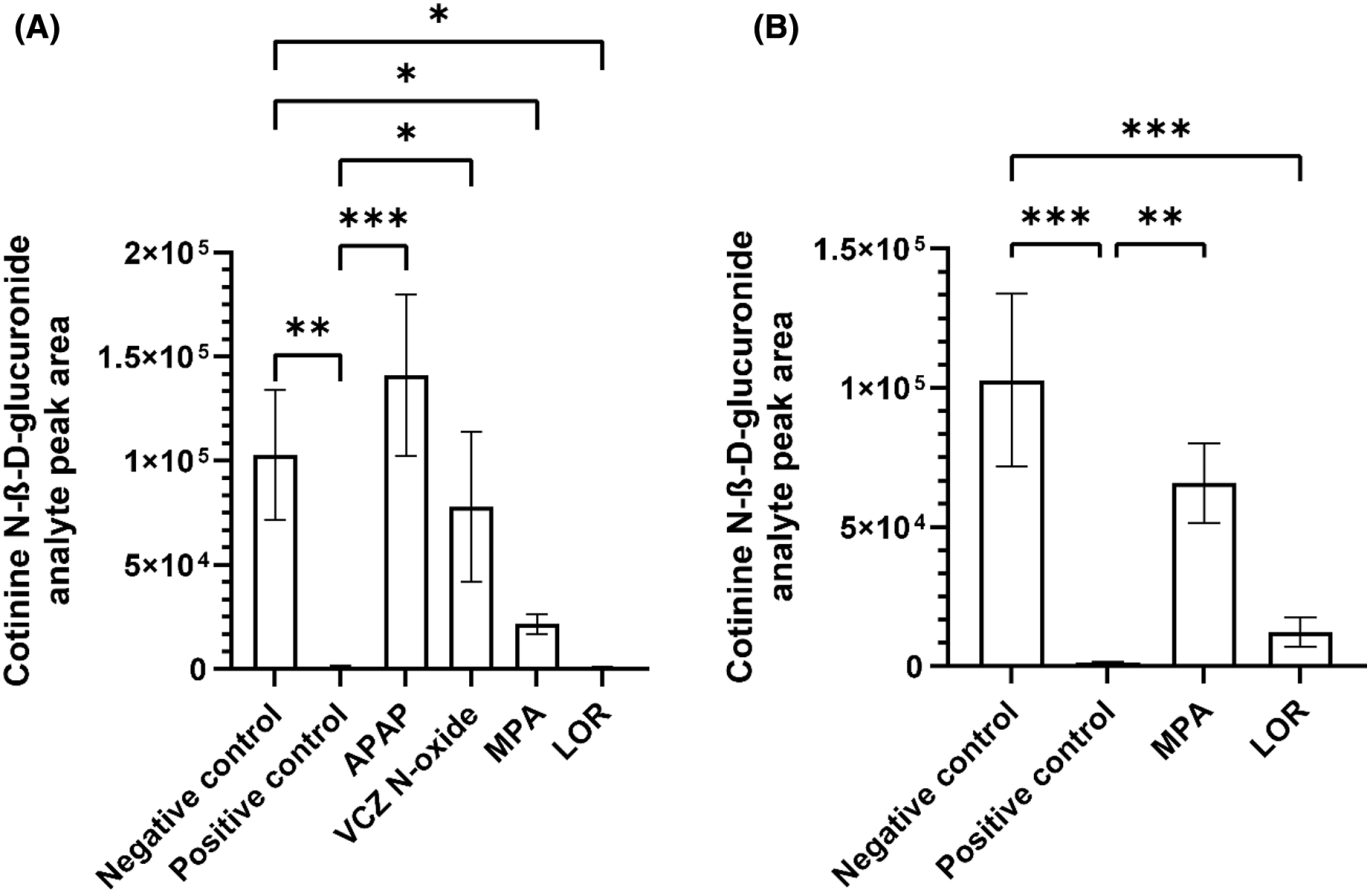
Screening of putative inhibitors of UGT2B10 enzyme at 5*C*_max_ and *C*_max_. (A) Each putative inhibitor was incubated at 5*C*_max_ with a fixed concentration of UDPGA (5 mM), UGT2B10 (0.5 mg/mL), and cotinine (4 mM) to measure enzyme activity. APAP (0.25 mM) and VCZ N‐oxide (0.075 mM) did not feature an inhibition in enzyme activity while MPA (0.4 mM) and LOR (0.15 μM) showed a significant reduction of 79% and 99.5% in enzyme activity, respectively. (B) MPA (0.08 mM) and LOR (0.03 μM) were incubated at *C*_max_ with a fixed concentration of UDPGA (5 mM), UGT2B10 (0.5 mg/mL), and cotinine (4 mM) to measure enzyme activity. Both molecules featured a reduction of 36 (nonsignificant) and 88% (significant) in enzyme activity, respectively. (A, B) In both graphs, positive control using AMT incubated at 10 μM in the same conditions showed an inhibition of 97.5% in enzyme activity. Negative control consisted of the absence of known/putative inhibitors and represented 100% of the activity. Each data point on the plot represents the mean of three replicates with SD. Statistical analysis was performed using ordinary one‐way ANOVA. *0.033 < adjusted *p*‐value <.050. **0.002 < adjusted *p*‐value <.033. ***Adjusted *p*‐value <.002.

**FIGURE 3:**
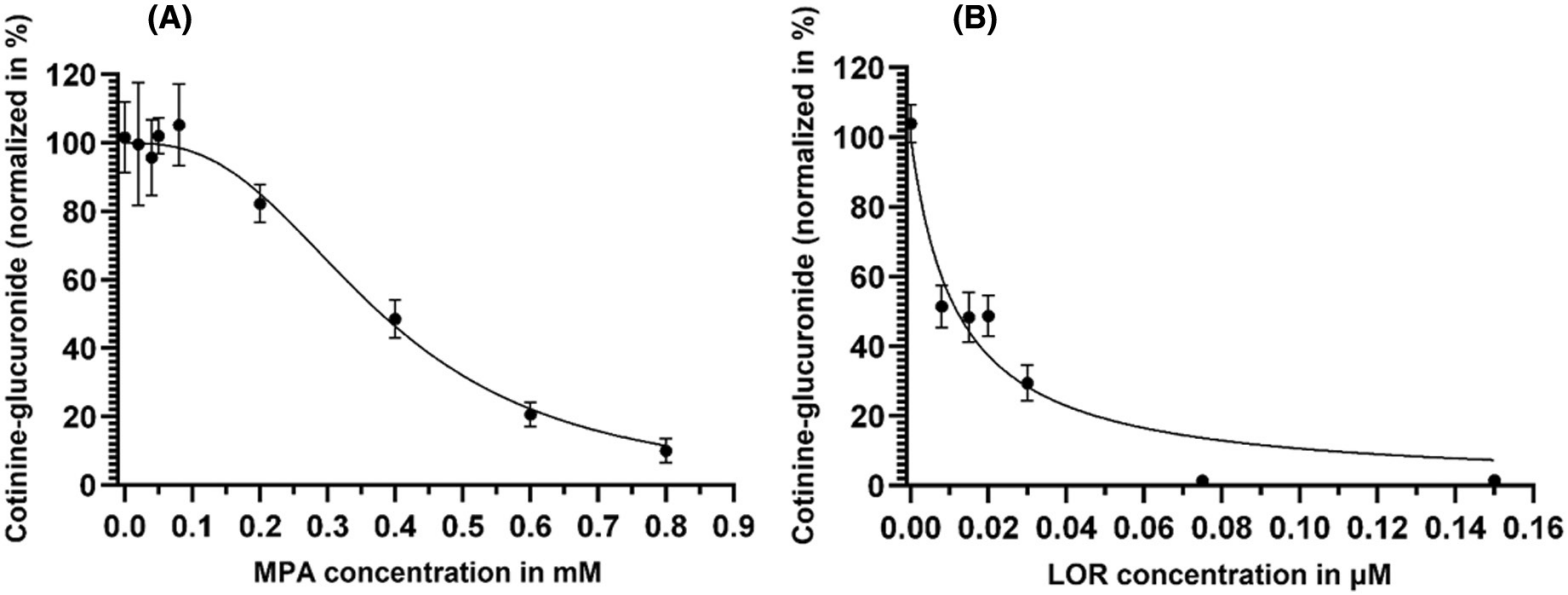
Effect of MPA and LOR on UGT2B10 enzyme activity. Addition of increasing concentrations of either MPA (A) or LOR (B), while keeping the same UDPGA (5 mM), UGT2B10 (0.5 mg/mL), and cotinine (4 mM) final concentrations were used to calculate the IC_50_ of both compounds for recombinant UGT2B10 with a nonlinear regression. IC_50_ was determined as 0.38 mM (*R* ^2^ = 0.9212) for MPA and 0.01 μM (*R* ^2^ = 0.9257) for LOR. Data points in both graphs represent the means of five replicates with SD. *X*‐axis represents log‐transformed concentrations of the molecules tested, *y*‐axis represents normalized cotinine glucuronide (%) considering cotinine glucuronide formation in the absence of inhibitor as 100%.

At 5 × *C*_max_, no inhibition of UGT2B10 was observed with APAP and VCZ N‐oxide(Figure 2A). On the other hand, a significant reduction of 79% and 99.5% in cotinine N‐ß‐d‐glucuronide formation was observed in the presence of MPA and LOR, respectively, compared to negative control (i.e., absence of perpetrator/inhibitor) (Figure 2A). At *C*_max_, these observed reductions were 36% (nonsignificant) and 88% (significant), respectively. Positive control with AMT showed an inhibition of 97.5% (Figure 2). IC_50_ values for MPA and LOR were determined as 0.38 mM and 0.01 μM, respectively (Figure 3).

### 3.4 Screening of UGT2B10 putative substrates

No glucuronidation of APAP, MPA, or LOR was observed at *C*_max_ and 10 × *C*_max_ upon incubation with UGT2B10. Incubations of LOR with UGT1A6 did not result in lorazepam‐glucuronide. However, incubations of APAP and MPA with UGT1A6 produced APAP‐glucuronide and MPA‐ß‐d‐glucuronide, respectively. Cotinine produced cotinine N‐ß‐D‐glucuronide upon incubation with UGT2B10 (positive control for instrumentation), but it was not metabolized by UGT1A6 (Figure S3). No glucuronides of VCZ N‐oxide and VCZ via UGT2B10 were detected using UHPLC‐HRMS. Detection of UGT1A4 mediated VCZ glucuronide formation (absence of chromatographic peak) was not observed either. VCZ peak intensity was not altered between negative control (absence of enzyme) with either UGT1A4 or UGT2B10 (Figure S4). Mean absolute peak intensities of VCZ are 9.29E+08 (RSD ± 6%), 9.28E+08 (RSD ± 10%), and 8.33E+08 (RSD ± 9%) in negative control, UGT1A4 and UGT2B10 enzymatic reactions, respectively. The mean obtained absolute peak intensity of VCZ in all samples is 9.05E+08 (RSD ± 8%). Similar to UHPLC–MS/MS, cotinine N‐ß‐D‐glucuronide was detected in UHPLC‐HRMS after incubation of cotinine with UGT2B10 (Figure S5).

Since no glucuronidation was observed with VCZ incubated with UGT1A4, midazolam was tested with UGT1A4 as a positive control at 70 μM (*K*_m_ of 64 μM)^5^ to rule out nonfunctional UGT1A4. Midazolam peak intensities were 2.47E+09 (RSD ± 8%) in the negative control and 1.65E+09 (RSD ± 7%) in the UGT1A4 reaction. This decrease in intensity indicates the occurrence of glucuronidation (Figure S6).

## 4 DISCUSSION

Among the prioritized ligands from the in silico approach, that is, APAP, LOR, MPA, and VCZ N‐oxide, we report for the first time LOR and MPA as UGT2B10 inhibitors in vitro at clinically relevant concentrations, even though they are not substrates of UGT2B10. Moreover, APAP and MPA are demonstrated to be substrates of UGT1A6 catalyzing O‐glucuronidation (Figure S4), with no other candidates tested being substrates for either UGT2B10 or UGT1A6. Both molecules were previously described as substrates of UGT1A6.^24^, ^25^, ^26^, ^27^ Finally, we observed no glucuronidation of VCZ by either UGT2B10 or UGT1A4 at clinical concentrations tested. On the contrary, midazolam is N‐glucuronidated by UGT1A4 when tested at its previously reported *K*_m_ concentration.^5^

LOR and MPA are used in HSCT setting for seizure prophylaxis and graft versus host disease prophylaxis, respectively.^28^, ^29^ In addition, LOR is also used in the treatment of anxiety as well as insomnia^30^, ^31^ and MPA is used as an immunosuppressant in many conditions, including solid organ transplantation.^32^, ^33^ (S)‐LOR is mainly glucuronidated by UGT2B15, followed by 2B4 and 2B7, whereas (R)‐LOR is additionally glucuronidated by 1A7 and 1A10.^4^, ^34^, ^35^ LOR is rarely associated with drug‐induced hepatotoxicity, with only one case reported^36^ (https://livertox.nih.gov). MPA is mainly (95%) metabolized by UGT1A9/1A8 into a 7‐O‐glucuronide and a smaller proportion through UGT2B7 into an acyl‐glucuronide.^2^, ^10^, ^26^, ^37^, ^38^ Similarly to LOR, hepatotoxicity cases induced by MPA are rare, with generally mild and easily reversible symptoms^39^ (https://livertox.nih.gov). In our study, both molecules were not found to be substrates of UGT2B10. UGT2B10 is homologous to 2B11 (89%), 2B7 (87%), 2B15 (85%), 2B17 (85%), and 2B4 (78%).^1^, ^40^ Their expression and activities feature a strong positive correlation with UGT2B10. In addition, UGT2B7, 2B15, and 2B4 reach their adult activity within 1‐month postbirth similar to UGT2B10.^2^, ^4^ It is possible for a substrate of one isoform to act as an inhibitor for another homologous isoform, even if it is not a substrate of the latter. This could be eventually observed when an isoform catalyzing the glucuronidation reactions of a ligand is less active or absent. UGT2B10 inhibition by LOR and MPA in our study may imply that they may inhibit UGT2B7, UGT2B15, or UGT2B4 considering their similarities to UGT2B10 in sequence, structure, and ontogeny of expression and activity.^1^, ^2^, ^4^

UGT2B10 was inhibited by LOR with an IC_50_ of 0.01 μM, which is threefold lower than its clinically observed *C*_max_. Further investigations are needed to understand the clinical relevance of this inhibition because it could modulate the UGT activity and metabolism of other co‐administered drugs. LOR was partly suggested to be a competitive inhibitor in our previous in silico investigation (Table 2),^11^ not supported by IC50 screening results, where no clear dose‐dependent inhibition was observed. The clinical relevance exploration of UGT2B10 inhibition by LOR in poor metabolizers of 2B15, 2B7, or 2B4 receiving another competing drug metabolized by these isoforms and/or UGT2B10 is feasible.^4^ MPA featured an inhibition of UGT2B10 at IC_50_ being fivefold higher than *C*_max_ and suggesting a competitive inhibition of UGT2B10 when considering that this was hypothesized in our in silico report (Table 2).^11^ Elucidating inhibitory constant (Ki) values for LOR, MPA, and relating them with IC50 values may reveal the exact nature of inhibition. UGT1A9 reaches its adult activity in infants (<2 years old) which explains why MPA glucuronidation was shown to be maximal before 4 months of age.^4^ It is likely that UGT1A9 poor metabolizers may have high MPA levels and can contribute to UGT2B10 inhibition, especially when the contributions of 2B7 as well as 1A6 are minor.^26^ The clinical relevance of UGT2B10 inhibition by MPA especially in relation to the assessment of drug–drug interactions is warranted.

**TABLE 2:** Status of prioritized molecules toward UGT2B10 following in silico investigation versus confirmed status following in vitro investigation.

Drug	APAP	VCZ N‐oxide	MPA	LOR	VCZ
In silico prediction	Substrate/Competitive Inhibitor	Substrate/Competitive Inhibitor	Competitive inhibitor/Substrate	Competitive inhibitor/Substrate	Substrate
In vitro testing (predefined concentrations)	Neither a substrate nor an inhibitor	Neither a substrate nor an inhibitor	Competitive inhibitor	Competitive inhibitor	Not a substrate

In this study, we reported glucuronidation of MPA by UGT1A6, which is consistent with previously described reports.^26^, ^27^ UGT1A6 features a slow maturation reaching its adult activity during adolescence (12–18 years old). Considering the role of UGT1A6 in the metabolism of MPA in addition of the major glucuronidation by UGT1A9,^4^, ^37^ it is likely that UGT1A6 plays a role in adolescents and adults. However, experiments determining the *K*_m_ of MPA for UGT1A6 are required to understand its role in adults and children in the abundance or dearth of UGT1A9.

An important aspect worthy to mention is that we based our analysis on the total *C*_max_ values of putative inhibitors and did not account for solely the unbound fraction, which is 8%–12% and 3% for LOR and MPA, respectively.^41^, ^42^ Indeed, the use of unbound *C*_max_ is based on the assumption that the drug's intracellular concentration is in thermodynamic equilibrium with the extracellular one.^43^ However, the correlation between the unbound plasma fraction and the fraction present in microsomes, which is a determining factor for drug metabolism and interactions, may not necessarily exist.^44^ As a matter of fact, when a treatment extends over multiple days, the drug may bind nonspecifically to liver microsomes and accumulate in liver cells, reaching concentrations likely higher than what might be expected based solely on unbound fraction in plasma.^45^ While being a common phenomenon for lipophilic compounds, no evidence of nonspecific binding of LOR and MPA to hepatic microsomes exists. However, models used in previous studies for MPA nonspecific binding assessment did not fully capture the complexity of in vivo biological processes, such as potential MPA's accumulation over time, intervariable enterohepatic cycling, and other individual physiological variations.^46^, ^47^, ^48^ Another aspect to account for is that unbound drug levels of MPA and LOR are known to fluctuate significantly (40%–50% of variability). In some conditions such as infections, reduced albumin levels, or impaired organ function, these levels can increase as high as five‐ to tenfold than the average values.^49^, ^50^, ^51^, ^52^ This variability is crucial for vulnerable patients taking multiple medications that undergo glucuronidation by homologous UGT enzymes (including UGT2B7, UGT2B15, and UGT2B10). In these cases, the inhibition of UGT2B10 may have an even more pronounced effect, potentially impacting drug metabolism and efficacy. Consequently, we assessed the status of our putative ligands with *C*_max_ for safety matters, and to generate the inhibitors IC50 that eventually can be used in physiologically based pharmacokinetic (PBPK) simulations for addressing the in vivo relevance of drug–drug interaction feasibility in clinic.

Among the other drugs tested in our experiments, APAP has a phenol ring prone to O‐glucuronidation in addition to secondary amine possibly N‐glucuronidated by UGT2B10.^1^, ^5^, ^11^ APAP is known to be glucuronidated through UGT1A1, UGT1A6, UGT1A9, and UGT2B15.^24^, ^25^ Incubation of APAP with UGT2B10 did not show any glucuronidation unlike with UGT1A6 where we observed glucuronide as reported earlier.^24^, ^25^ In addition, APAP did not inhibit UGT2B10 in our study and it is not known to inhibit any other UGT isoform. These results are inconsistent with our in silico predictions on APAP (Table 2).^11^ Another candidate, VCZ, a commonly prescribed antifungal agent in HSCT, is known to be hepatotoxic.^53^ VCZ is metabolized into VCZ N‐oxide by CYP2C19 and was also described to form VCZ N‐glucuronide by UGT1A4 without going through a phase I metabolism intermediate.^5^, ^54^, ^55^ VCZ (triazole) N‐glucuronidation by UGT1A4 is minimal at clinical concentrations (*K*_m_ = 1.130 mM) compared to imidazole‐containing antifungals (e.g., bifonazole; *K*_m_ = 28 μM).^5^ Though UGT1A4 activity is variable, adults' activity is reached between 1 month and 1 year during infancy, hence assessing VCZ metabolism through this pathway is warranted.^4^ UGT1A4 also shares substrates with UGT2B10 as both participate in N‐glucuronidation.^7^, ^11^, ^19^, ^56^ We did not observe any direct N‐glucuronidation of VCZ at clinically observed concentrations through UGT2B10 or UGT1A4. In our experiments, we used concentrations that were 25‐fold lower than those tested in Bourcier et al. study that reported direct VCZ N‐glucuronidation by 1A4.^5^ Considering the structural proximity of midazolam with VCZ,^2^, ^4^, ^5^ we used it as a positive control for incubations with UGT1A4 at its *K*_m_ concentrations and it featured glucuronidation in our experiments. Azole antifungals are also known to inhibit UGT2B7.^13^, ^57^, ^58^, ^59^ However, inhibition is apparent with imidazole‐containing antifungals such as ketoconazole and econazole than triazole‐containing antifungals such as voriconazole and fluconazole.^5^ The in silico screening performed previously^11^ predicted the status of VCZ N‐oxide as a competitive inhibitor of UGT2B10, but this was not the case in our experiment, suggesting few false positives in in silico screening as observed with other molecules (Table 2). However, it does not alleviate the utility of this approach for prioritization of molecules for in vitro testing.

To conclude, LOR and MPA were reported as UGT2B10 inhibitors at clinical concentrations, warranting assessment of the clinical relevance of this inhibition. We also established a pipeline combining highly selective, sensitive, and validated analytical method to screen in vitro transformation of putative ligands by UGT2B10 following prioritization by in silico screening. This approach highlighted the critical importance of obtaining inhibition data from in vitro studies on similar enzymes, which can be applied in developing accurate PBPK models to predict in vivo drug–drug interactions. This is particularly relevant when a single compound can inhibit multiple enzymes at clinically observed concentrations, highlighting the fact that the complexity of drug–drug interactions is significant when involving glucuronidation enzymes and requires a more comprehensive approach to prediction and analysis.

## AUTHOR CONTRIBUTIONS

*Participated in research design*: Uppugunduri, Daali, Ben Hassine, Ansari. *Conducted experiments*: Gencaslan, B. Khoshbeen, Bennani. *Contributed new reagents or analytic tools*: Daali, Bennani, Gencaslan, Ben Hassine, Ansari. *Performed data analysis*: Gencaslan, Bennani, Ben Hassine,Uppugunduri. *Wrote or contributed to the writing of the manuscript draft*: Bennani, Uppugunduri, Gencaslan, Ben Hassine, Daali, Ansari, Samer.

## FUNDING INFORMATION

This research work was supported by the CANSEARCH Foundation and the OAK Foundation (PI: Marc Ansari; grant number: OCAY‐17‐642).

## CONFLICT OF INTEREST STATEMENT

The authors declare no conflict of interest.

### OPEN RESEARCH BADGES

This article has earned Open Data, Open Materials and Preregistered Research Design badges. Data, materials and the preregistered design and analysis plan are available at [[insert provided URL(s) on the Open Research Disclosure Form]].

## Supporting information


**Data S1:** Supporting Information.

## Data Availability

The authors declare that all the data supporting the findings of this study are available within the paper, its supplemental data, and from the Yareta repository (https://doi.org/10.26037/yareta:4f2kuon35jc27opnijubgqkmge).
