# Supplementary material for: In vitro screening of UGT2B10 in silico prioritized putative ligands from drugs used in the pediatric hematopoietic stem cell transplantation setting
Source: Pharmacol Res Perspect. 2024 Nov 29;12(6):e70011. doi: 10.1002/prp2.70011 (PMC11605732; doi:10.1002/prp2.70011)

**Supplementary Materials**

In vitro screening of UGT2B10 in silico prioritized putative ligands from drugs used in the pediatric hematopoietic stem cell transplantation setting

Yahia BENNANI, Khalil BEN HASSINE, Muhammed GENCASLAN, Mary BOUDAL-KHOSHBEEN, Tiago NAVA, Caroline SAMER, Marc ANSARI, Youssef DAALI, Chakradhara Rao Satyanarayana UPPUGUNDURI

Division of Clinical Pharmacology and Toxicology, Geneva University Hospitals, Geneva, Switzerland (Y.B., C.S., Y.D.)

Geneva Lausanne School of Pharmacy, University of Geneva, Geneva, Switzerland (Y.B., M.G., C.S., Y.D.)

Swiss Center for Applied Human Toxicology, Geneva, Switzerland (C.S., Y.D.)

Faculty of Medicine, University of Geneva, Geneva, Switzerland (C.S., Y.D.)

CANSEARCH Research Platform in Pediatric Oncology and Hematology, Department of Pediatrics, Gynecology and Obstetrics, University of Geneva, Geneva, Switzerland (K.B.H., M.B.K., M.A., C.R.S.U.)

Division of Pediatric Oncology and Hematology, Department of Pediatrics, Gynecology and Obstetrics, Geneva University Hospitals and University of Geneva, Geneva, Switzerland (M.A)

**Corresponding author**

Dr. Chakradhara Rao Satyanarayana Uppugunduri, Ph.D.

CANSEARCH Research Platform in Pediatric Oncology and Hematology and Division of Pediatric Oncology and Hematology, Department of Pediatrics, Gynecology and Obstetrics, Geneva University Hospitals and Faculty of Medicine of University of Geneva, 64 Avenue de la Roseraie, CH-1205, Geneva, Switzerland

Tel: 41-78-696-96-73

Fax: 41-22-382-31-00

E-mail address: [chakradhara.uppugunduri@unige.ch](mailto:chakradhara.uppugunduri@unige.ch)

**Supplementary Text S1. Validation procedure of the analytical method for cotinine N-ß-D-glucuronide quantification.**

**Selectivity and carry-over**

The selectivity of the method was measured by checking the absence of interfering peaks at compound retention time (RT) for cotinine N-ß-D-glucuronide and the internal standard (IS) morphine-3-ß-glucuronide. The carry-over effect was determined for each analyte by analyzing the highest calibration standard solution followed by the analysis of the blank.

**Linearity, accuracy and precision**

For each of three non-consecutive days, the linearity of the assay was assessed by duplicate analysis of calibrators (n = 2) with concentrations of 10, 20, 50, 100, 200, 500 and 1000 ng/mL for cotinine N-ß-D-glucuronide. These seven solutions were prepared by adding 10 µl of 50, 100, 250, 500, 1000, 2500 and 5000 ng/mL stock solutions respectively to 10 µl of tris-HCl buffer at 100 mM of pH 7.4, 5 µl of MgCl_2_ at 50 mM, 25 µl of UDPGA at 10 mM and 100 μl of acetonitrile containing 675 ng/mL of Mo-3-G. Quantitation was performed by plotting the ratios of the peak areas of cotinine N-ß-D-glucuronide to the IS versus concentrations.

Accuracy and precision (repeatability and intermediate precision) were assessed during the same three non-consecutive days by three different QC samples of two replicates spiked with IS. The QC concentrations were 20, 100 and 500 ng/mL prepared with the same procedure as for calibrators and using stock solutions at 100, 500 and 2500 ng/mL. The QC samples covered the expected range of cotinine N-ß-D-glucuronide concentration in the experimental assays.

**Stability**

Stability experiments were conducted on five different incubation samples corresponding to five different incubation times (10, 20, 30, 90 and 120 minutes) in duplicates. These samples were stored at -20°C for 6 days as it is the usual storage condition for such samples. The initial mean of the area under the curve (AUC) of cotinine N-ß-D-glucuronide obtained after each incubation time was compared with AUC of the analyte after the storage.

**Supplementary Text S2. Validation results of the analytical method for cotinine N-ß-D-glucuronide quantification.**

**Selectivity and carry-over**

No significant residual signals were observed when analyzing blank samples at the retention times of cotinine N-ß-D-glucuronide and IS. In addition, precaution was always taken by injecting a blank solution between different concentrations of the solutions to avoid any potential carry-over effect.

**Linearity, accuracy and precision**

Plotting the peak area ratio cotinine N-ß-D-glucuronide/IS against the analyte concentration provided a seven-point calibration curve. A linear regression model using a 1/x^2^ weighting factor was used as a fit. The correlation coefficients of the calibration curve for each of the three non-consecutive days were over 0.990, proving a linear relationship.

The obtained accuracy data are 91.5-111.8 % for cotinine N-ß-D-glucuronide and within the acceptance criteria range (80–120 % of the theoretical value). Precision values (repeatability and intermediate precision) for the three QC samples are 8.5–11.0 % and within the acceptance range, in accordance with the guidelines (under 20 %). The individual values of accuracy and precision can be seen in the following table:

| QC concentration (ng/mL) | Accuracy (%) | Precision | |
| --- | --- | --- | --- |
|  |  | Repeatability (RSD%) | Intermediate precision (RSD%) |
| 20 | 91,50% | 8,50% | 8,50% |
| 100 | 93,60% | 9,90% | 9,90% |
| 500 | 111,80% | 11,00% | 11,00% |

**Stability**

| Incubation time | Initial AUC | Final AUC | Ratio (final/initial) |
| --- | --- | --- | --- |
| 10 | 2,77E+04 | 3,03E+04 | 91% |
| 20 | 4,76E+04 | 4,80E+04 | 99% |
| 30 | 4,92E+04 | 5,04E+04 | 98% |
| 90 | 2,03E+05 | 1,69E+05 | 120% |
| 120 | 1,35E+05 | 1,58E+05 | 85% |

Stability data are summarized in the following table:

All the samples were stable and didn’t show a significant degradation by staying within the acceptance range of 20%.

| **Compound** | **Concentration of stock solution** | **Solvent** |
| --- | --- | --- |
| Acetaminophen | 20 mM | Milli Q water |
| Acetaminophen-glucuronide | 5 mg/ml | Methanol |
| Mycophenolic acid | 20 mM | Acetonitrile |
| Mycophenolic acid-ß-D glucuronide | 1 mg/ml | Acetonitrile: Methanol (50:50) |
| Lorazepam | 3.11 mM | Acetonitrile |
| Lorazepam-glucuronide | 100 ug/ml | Acetonitrile: Methanol (50:50) |
| Voriconazole | 5 mg/ml | Dimethyl sulfoxide |
| Voriconazole N-oxide | 6 mM | Dimethyl sulfoxide |
| Amitriptyline | 1 mM | Milli Q water |
| Cotinine | 50 mM | ACN: Methanol |
| Cotinine N-ß-D-glucuronide | 8 mM | Milli Q water |
| Morphine-3-ß-glucuronide (Mo-3-G) | 1 mg/ml | Dimethyl sulfoxide |
| Recombinant UGT2B10 | 5 mg/ml | - |
| Recombinant UGT1A6 | 5 mg/ml | - |
| Recombinant UGT1A4 | 5 mg/ml | - |
| Tris-HCl buffer | 100 mM of pH 7,4 | - |
| UDPGA | 10 mM | Tris-HCl buffer |
| MgCl_2_ | 50 mM | Milli Q water |
| Midazolam | 1 mg/ml | Methanol |

**Supplementary Table S1.** **Concentration of stock solutions and solvents of the study compounds.**

| **Compounds** | **Q1 m/z (Da)** | **Q3 m/z (Da)** | **Declustering Potential** | **Collision Energy** | **Collision Cell Exit Potential** |
| --- | --- | --- | --- | --- | --- |
| Cotinine N-ß-D-glucuronide | 353.401 | 177.400 | 91 | 25 | 20 |
| Mo-3-G | 462.200 | 286.300 | 71 | 43 | 18 |
| LOR-glucuronide | 497.339 | 321.200 | 71 | 19 | 26 |
| MPA-ß-D-glucuronide | 497.419 | 303.300 | 51 | 21 | 22 |
| APAP-glucuronide | 328.466 | 152.400 | 66 | 13 | 16 |
| VCZ N-oxide-glucuronide | No glucuronide metabolite available on the market | | | | |

**Supplementary Table S2. Mass transitions of detected compound in UHPLC-MS/MS.**

**Supplementary Figure S1. Chromatograms of MPA-ß-D-glucuronide, LOR-glucuronide, APAP-glucuronide and cotinine N-ß-D-glucuronide standards**.

**
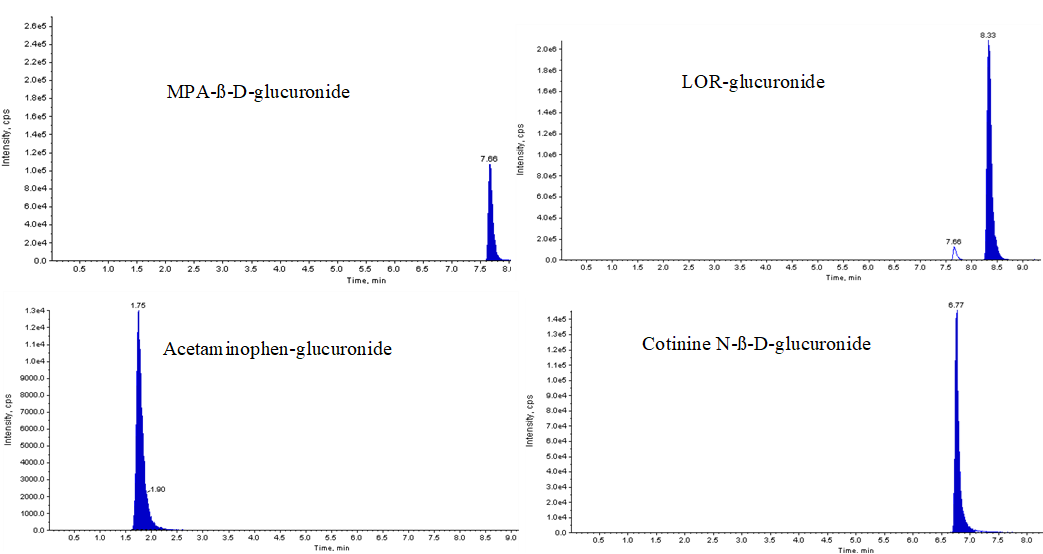
**

**Supplementary Figure S2. Effect of incubation time and DMSO proportion on UGT2B10 enzyme activity. (A) Increasing incubation time whilst keeping the same UDPGA (5 mM), UGT2B10 (0.5 mg/mL) and cotinine (5 mM) final concentrations was used to measure enzyme activity. Optimal incubation time was determined as 90 minutes. (B) Increasing DMSO proportion whilst keeping the same UDPGA (5 mM), UGT2B10 (0.5 mg/mL) and cotinine (5 mM) final concentrations was used to measure enzyme activity. Optimal DMSO proportion was determined as 1 % or less. Data points in graph A and graph B represent the means of two and three replicates with SD, respectively.**

**
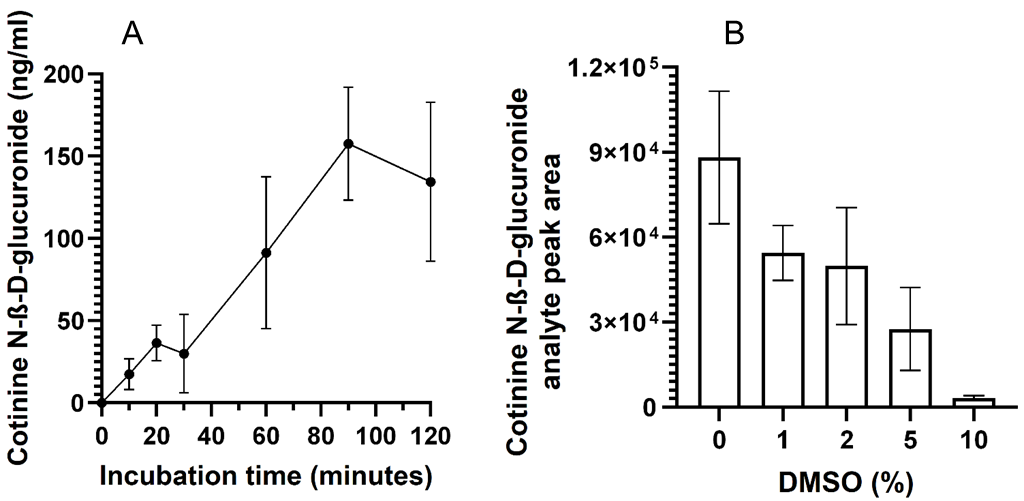
**

**Supplementary Figure S3. Chromatograms resulting from incubations of cotinine with UGT2B10 as well as APAP and MPA with UGT1A6 after injection in UHPLC-MS/MS.**

**
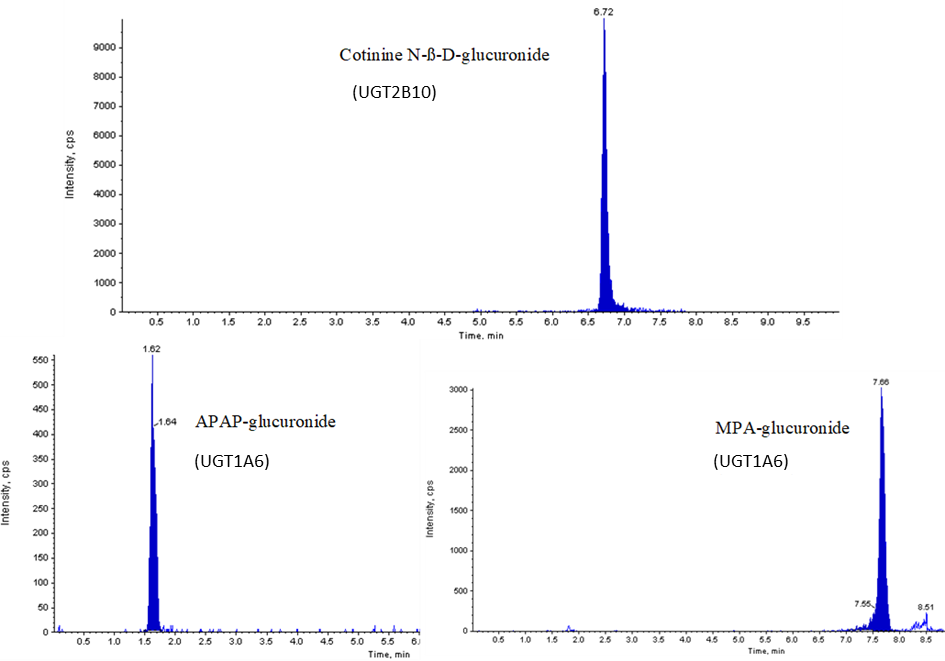
**

**Supplementary Figure S4. Chromatogram resulting from incubations of VCZ with UGT1A4 after injection in UHPLC-HRMS. The same peak was obtained with negative control (absence of enzyme) and UGT2B10 incubation in three replicates**.

**
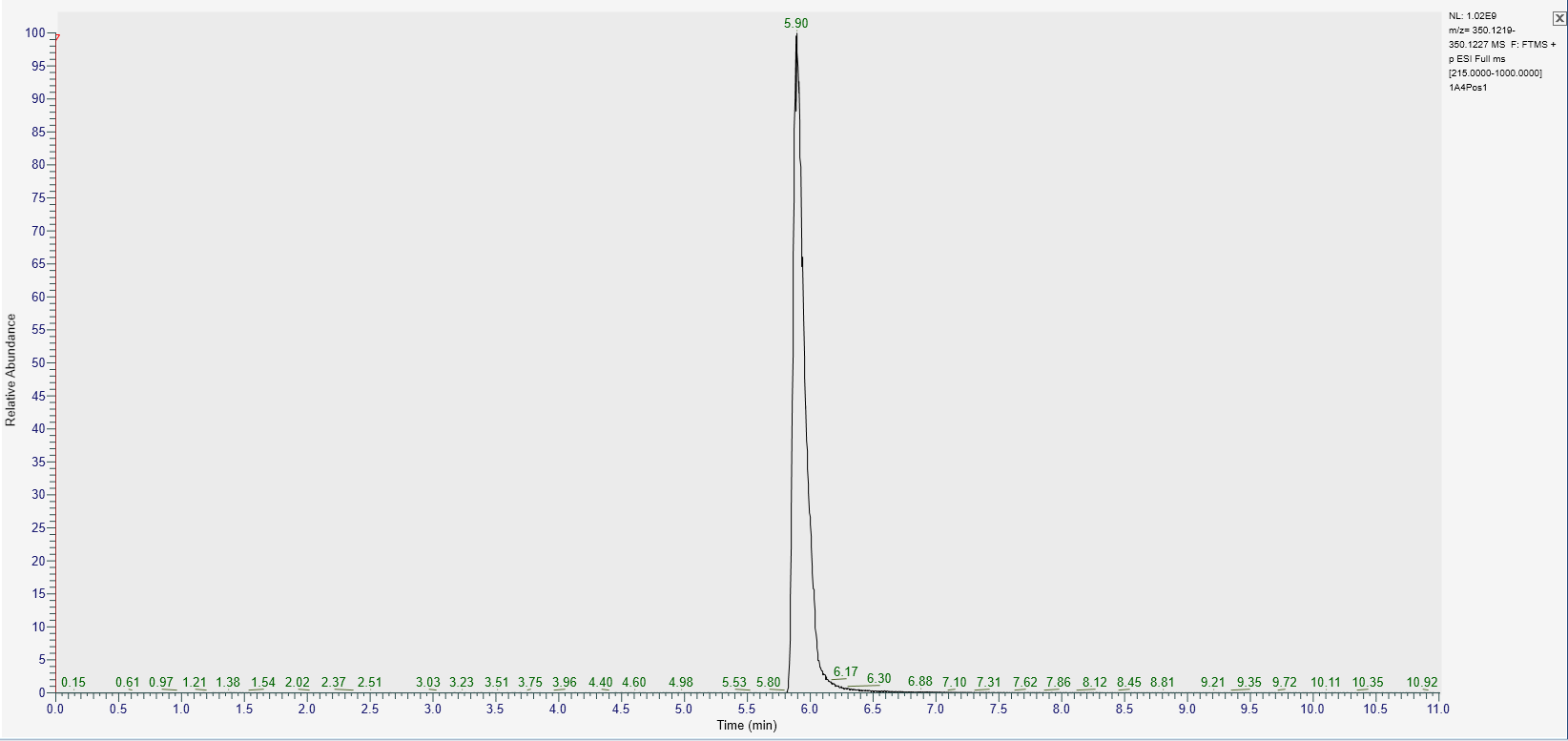
**


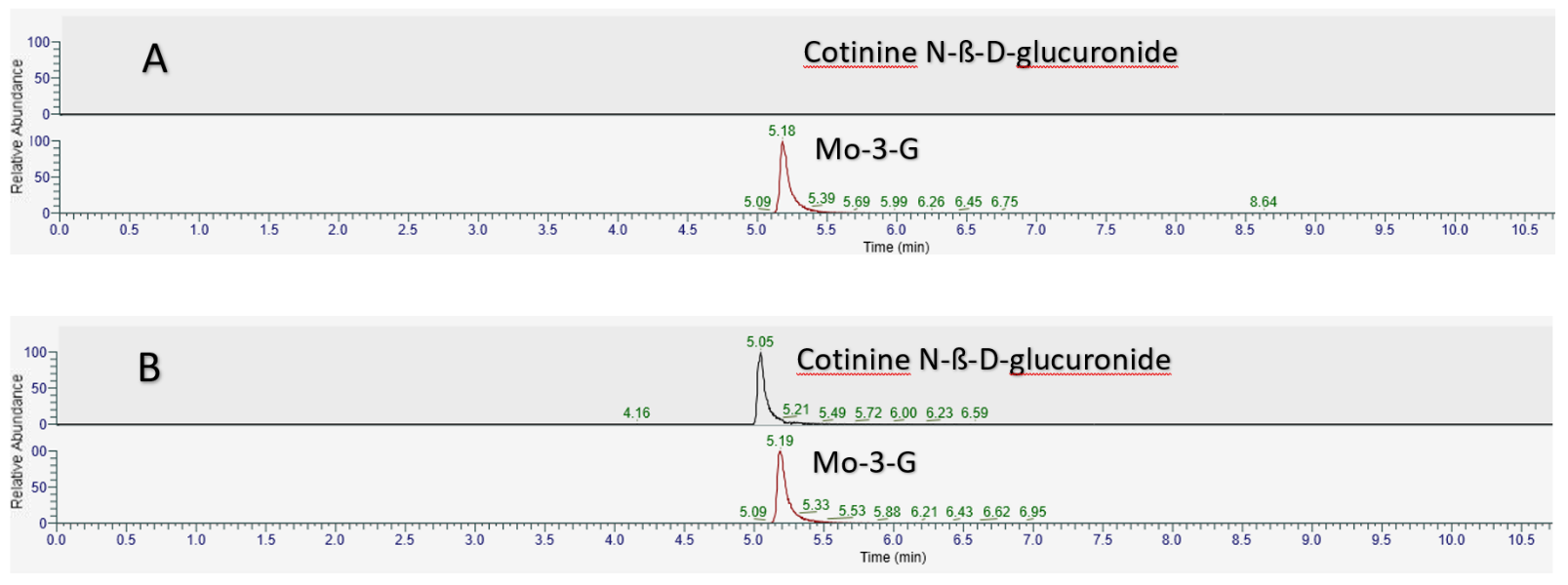
**Supplementary Figure S5. Chromatograms resulting from incubations of cotinine without (A) and with (B) UGT2B10 after injection in UHPLC-HRMS. Mo-3-G was also analyzed as a control detection.**

**Supplementary Figure S6. Chromatograms resulting from incubations of midazolam without (A) and with (B) UGT1A4 after injection in UHPLC-HRMS**.


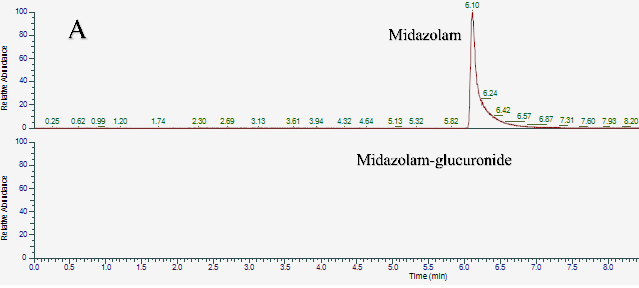

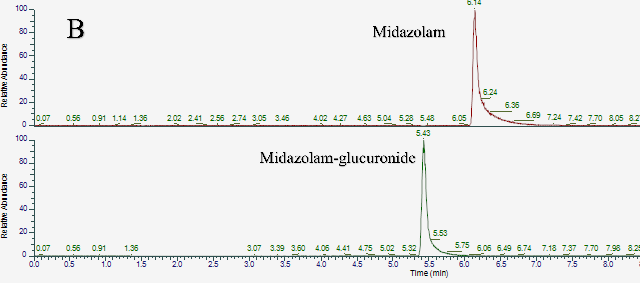

Supplement: Supplementary file 1 — Data S1: Supporting Information. [file PRP2-12-e70011-s001.docx]
